# Immigration and Education

**Published:** 1885-04

**Authors:** J. B. Chandler


### IMMIGRATION AND EDUCATION.

J. B. CHANDLER.

The citizen of a Republic needs constantly to bear in mind that the price of Liberty is eternal vigilance. To him public affairs are personal affairs; he may be but a unit in a large sum, yet that sum is *supposed* to be made up of units that think and act; and the preservation of the institutions of the country depend upon the proper thought and action of a majority of units.

Senates and Houses of Representatives should be merely the medium of carrying out the wishes of the people as expressed on the questions of the hour through the ballot box.

Public opinion *should be* the result of calm, dispassionate consideration of the subject, aided by discussion of the pros and cons by able arguers on both sides, either through the public press or in any other way most likely to reach the general eye and ear.

Then, given a population made up of clear minded educated people, all looking toward one end, the general good and the preservation of institutions of the Republic, the result could scarcely be doubtful.

But, unfortunately, there are discordant elements which, unless controlled, may drive the Ship of State upon the quicksands of anarchy and ruin.

There are questions of great importance to the welfare of the people constantly arising, many of which are seized upon by political parties and are fully ventilated in party organs and from the stump, to the delectation of admiring crowds of the orators' friends; but the quiet, dispassionate consideration of their merits is too often lost in the desire for party policy and party success.

Groups of "silver men;" dynamite advocates; sectarian bigots; trade combinations; corporations; grangers; fiat money advocates, &c., &c., *ad infinitum*, are constantly at work; each of which ignoring all other interests is busy tearing down, undermining or building up for its especial profit and advancement.

Some of these groups may be advocating what is really for the general good; but what is to be of general effect is of interest to each individual who has cast his lot amongst us. Just here arises the question: What is to make the individual capable of discerning right from wrong or indeed of appreciating the bearings of any question upon the institutions of our government; especially if that individual has spent the greater part of his life in poverty, ignorance and blind superstitious obedience, under a form of government different from this.

That question it was thought was met by the establishment of public schools, which even if enforced attendance should be necessary, must eventuate in the education of, at least, the children of immigrants, and it was generally conceded that through the enlightenment of liberal education, poverty, ignorance and superstition must be eradicated.

In point of fact up to a certain time this proved true, and many of our notable men of the present are the offspring of those who sought a home here from foreign lands and gave their children the advantages of liberal provision made for public education and enlightenment. But of late the outpouring of emigration from Europe to this country has been of such a character and in such quantity that the means of adaptation, the avenues of purification have been almost clogged. Instead of immigrants being absorbed and united homogeneously with the general population, we find them in national groups keeping up the warfare and strife of the fatherlands and instead of adding to the general prosperity of their adopted country, they use the freedom which they cannot appreciate, to disturb us with crimes they meditated but dared not commit at home.

There are many who come with good intentions; induced by the success of honest industrious working friends and relatives who have preceded them; but even these now are discordant elements; the avenues of labor are crowded and the workingmen are combined to keep up wages at a standard which the state of trade, the rule of supply and demand, will not warrant; the result is that competition is to be met by employes as well as employers and the immigrant who would rather work than starve is the factor which, with the idle blatherskites who prevent settlements which could easily be made by quiet discussion between those directly interested, must eventually, if immigration is unchecked, place the workingmen of this country on the same plane as those of Europe. God forbid!

Perhaps I may have suggested one question worthy of calm consideration:

Is it not about time to consider whether a check had not better be put upon immigration from Europe as well as from Asia? At least as to quantity and quality.

I have stated that until the tide of immigration set in too strong, the results of liberal public education were all that had been expected; children graduated from the schools with the foundation laid for good citizenship; contentment and prosperity sealed the love of the immigrant and his family for their new home; a remembrance of the fatherland and early pleasures and sufferings might exist, but the land of adoption, its institutions once understood and appreciated, offered too many attractions and soon obliterated all desire to return.

Just here another question urges itself into notice, one that may be of great disturbing force before it is settled if not firmly met and in season:

Religious toleration is something dear to every American, but is not always, perhaps, provocative of general good, as is witnessed in Utah. Yet it is one of the cardinal points of our country and one to which it owes much, if not all, of its wonderful prosperity; yet when sectarianism, in the guise of religion, strikes at the root of a great public blessing and necessity, is it not time to inquire, whether a little toleration is not also due from sects to the general good? The Mormon pleads religion as his warrant for disobeying the laws of the country and founds his claim upon constitutional guarantee. What is equity in his case? Must the general good suffer for his personal belief? .

Again, we hear rumors of parents being refused the rights of their church, if they send their children to public schools or do not send them to schools in which the teachers or directors are mostly foreigners whose training has been not with the view of fitting them to instruct children in the great liberal principles and knowledge of a citizen of a free Republic, but rather to continue in the blind reliance upon others for their guide in life, and to the especial glorification of their particular sect and its authorities.

I do not wish to be understood as condemning any form of worship which has Almighty God as its object; nor would I condemn any reasonable efforts for propagating faith, but it does appear that our public schools are to lose a large portion of their usefulness if the children of immigrants who need them most, are to be deprived of their influence and given a sort of *lucus a non lucendo* education, which may eventually make them worse citizens, than would the ignorance which their parents were raised in. Education in which truth is suppressed or garbled will not make good citizens.

The foundation of a republican form of government is a bond of unity of its citizens; any thing that aims to break this up, to draw an unmistakable line between the people strikes at the root of all public and social happiness. Is it not possible for us to get along in Christian brotherly love, especially where religion is concerned, without jarring over the histories of the past? They contain unfortunately too much to be regretted by all sects. Let the children know it all, the good and the bad and perhaps when they grow up they will find out of t e past, lessons of profit for the future.

Whilst writing this article my eye has been attracted to a paragraph in the inaugural address of President Cleveland, it is so singularly apropos that I repeat it:

"The constitution which prescribes his oath " my countrymen is yours; the government you " have chosen him to administer is yours; the " suffrage which executes the will of freemen " is yours; the laws and the entire scheme of " our civil rule, from the town meeting to the " state capitals and the national capitol, is " yours. Your every voter as surely as your " chief magistrate under the same high sanc- " tion, though in different sphere, exercises a " public trust."

